# Supplementary material for: The Evolution and Characterization of the RNA Interference Pathways in Lophotrochozoa
Source: Genome Biol Evol. 2024 May 7;16(5):evae098. doi: 10.1093/gbe/evae098 (PMC11114477; doi:10.1093/gbe/evae098)
Supplement: evae098_Supplementary_Data [file evae098_supplementary_data.zip › Supplementary_materials.zip!/selected_miRNAs/NoGe_selected_miRNAs/mirdeep_pdf_files/NMRB01000697.1_Notospermus_geniculatus_strain_Ushimado_scaffold697,_whole_genome_shotgun_sequence_18912.pdf]

Provisional ID	:	NMRB01000697.1_Notospermus_geniculatus_strain_Ushimado_scaffold697,_whole_genome_shotgun_sequence_18912
Score total	:	49.3
Score for star read(s)	:	3.9
Score for read counts	:	44.5
Score for mfe	:	1.5
Score for randfold	:	0
Score for cons. seed	:	-0.6
Total read count	:	99
Mature read count	:	59
Loop read count	:	0
Star read count	:	40

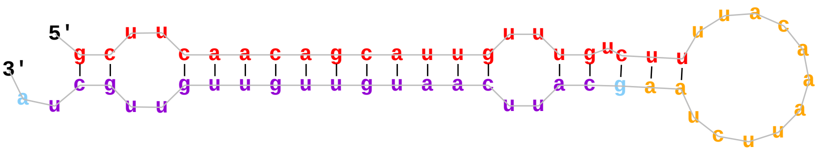

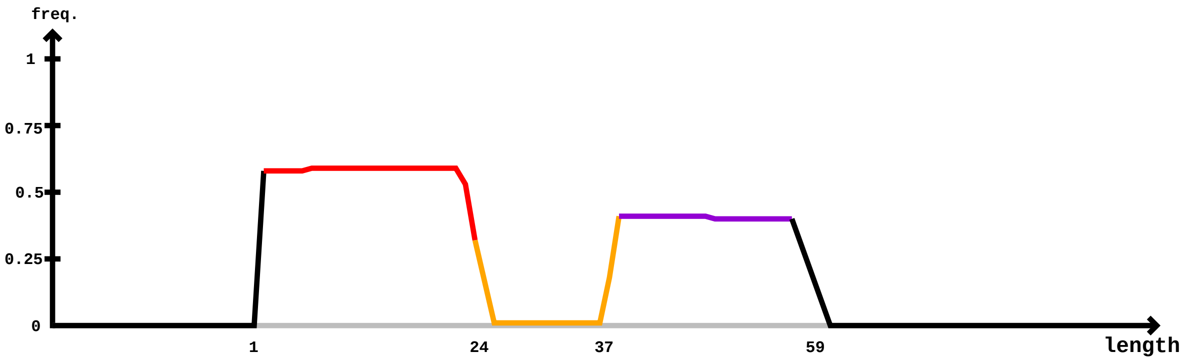


```
                   Mature                                 Star
5'- gaaaagaaaucucagccuuugcuucaacagcauuguuugucuuuuacaaaauucuaagcauucaauguuguuguugcuaaggacugggacaaaagucaacugaagaugaccccc   -3'   obs
    gaaaagaaaucucagccuuugcuucaacagcauuguuugucuuuuacaaaauucuaagcauucaauguuguuguugcuaaggacugggacaaaagucaacugaagaugaccccc         exp
    .........((((((((((.((..(((((((((((..((.(((...........)))))..)))))))))))..)).)))).))))))......((((........))))....   reads   mm   sample
    ..........................................................cauucaauguuguuguugcu....................................   4       0    Fm3

    ....................gcuucaacGgcauuguuuguc.........................................................................   6       1    Fm2
    ....................gcuucaacGgcauuguuugucu........................................................................   6       1    Fm2
    ....................gcuucaacGgcauuguuugucuu.......................................................................   9       1    Fm2
    ..........................................................cauucaauguuguuguugcua...................................   2       0    Fm2

    ....................gcuucaacagcauuguuugucu........................................................................   1       0    Fm1
    ....................gcuucaacagcauuguuugucG........................................................................   1       1    Fm1
    ....................gcuucaacagcauuguuugucuu.......................................................................   6       0    Fm1
    ....................gcuucaacagcauuguuugucuG.......................................................................   1       1    Fm1
    .........................................................gcauucaauguuguuguugcua...................................   4       0    Fm1

    ....................gcuucaacagcauuguuugucu........................................................................   1       0    Mm3
    .........................................................gcauucaauguuguuguugcu....................................   1       0    Mm3
    ..........................................................cauucaauguuguuguugcu....................................   2       0    Mm3
    ..........................................................cauucaauguuguuguugcuaU..................................   1       1    Mm3

    ....................gcuucaacGgcauuguuugucu........................................................................   2       1    Mm1

    ....................gcuucaacagcGuuguuugucu........................................................................   1       1    Mm2
    ....................gcuucaacagcauuguuugucu........................................................................   9       0    Mm2
    ....................gcuucaacagcauuguuugucuu.......................................................................   13      0    Mm2
    ....................gcuucaacagcauuguuugucuuu......................................................................   2       0    Mm2
    .........................aacagcauuguuugucuu.......................................................................   1       0    Mm2
    ...........................................uuacaaaauucuaagcauucaaugu..............................................   1       0    Mm2
    .........................................................gcauucaauguuguuguugcu....................................   8       0    Mm2
    .........................................................gcauucaauguuguuguugcua...................................   3       0    Mm2
    .........................................................gcauucaauguuguuguugcuaU..................................   1       1    Mm2
    ..........................................................cauucaauguuguuguGgcu....................................   1       1    Mm2
    ..........................................................cauucaauguuguuguugcu....................................   12      0    Mm2
    ..........................................................cauucaauguuguuguugcua...................................   1       0    Mm2
```
